# Stress recovery and restorative effects of viewing different types of peony garden landscapes: a preliminary experiment

**DOI:** 10.3389/fpsyg.2026.1935645

**Published:** 2026-09-28

**Authors:** Renlin Zhao, Yun Ma, Wenzhong Zhang, Xingzhe Hu, Xinping Zhang

**Affiliations:** 1 College of Urban Planning and Municipal Engineering, Xi’ an Polytechnic University, Xi’an, China; 2 College of Landscape Architecture and Art Design, Hunan Agricultural University, Changsha, Hunan, China; 3 School of Art and Design, Xi’an University of Technology, Xi’an, China

**Keywords:** physiological feedback, psychological scale, specialized garden, stress recovery, tree peony, virtual reality

## Abstract

**Background:**

Substantial evidence supports the health benefits of urban green spaces, yet most studies focus on general landscapes, leaving a gap regarding specialized gardens with topographic variations (flatland, tableland, mountain land). This study explored the restorative effects of peony garden scenes with different topographic characteristics on students’ physical and mental health using virtual reality (VR) and the Trier Social Stress Test (TSST).

**Methods:**

Participants (*N* = 56 per group, 23 ± 1.5 years old) were exposed to flatland peony garden (FPG), terraced peony garden (TPG), mountainous peony garden (MPG), or indoor environment (IE). Physiological factors were examined using heart rate (HR), heart rate variability (HRV), and electroencephalogram (EEG). psychological evaluation was carried out using the Profile of Mood States (POMS) and State–Trait Anxiety Inventory (STAI). Perceived Restorativeness Scale (PRS) was used to assess subjective perceived recovery.

**Results:**

Results showed that: (1) After TSST, HR, LF/HF, and Beta significantly increased (*p* < 0.01), while R-R, HF, and Alpha decreased (*p* < 0.01); (2) Compared to the stress stage, viewing FPG, TPG, and MPG led to significant physiological changes (*p* < 0.05), increased positive emotion (V-A, *p* < 0.01), and decreased negative emotions (A-H, F-I, T-A, C-B, D-D, anxiety, *p* < 0.01); (3) Perceived recovery was associated with emotional states, reflected in greater feelings of “being away” and “fascination.” The most pronounced changes occurred in the MPG.

**Conclusion:**

In the current VR experiment, peony garden landscapes, especially those with abundant landscape elements and visual experience, exhibited therapeutic potential for short-term stress recovery compared with the windowless indoor office scene. The findings offer preliminary guidance for creating health-promoting peony garden landscapes.

## 1 Introduction

The environmental stressors and mental health challenges posed by rapid urbanization are intensifying (Liu L. et al., 2022). Green space landscapes and their restorative effects on physical and mental health have become a key research focus in environmental psychology and landscape planning (Deng et al., 2020). Numerous empirical studies showed that exposure to green environments facilitates physiological stress recovery and attention restoration (Liu L. et al., 2022; Deng et al., 2020; Cao et al., 2024; Malekinezhad et al., 2020). Restorative benefits vary by landscape types (Cao et al., 2024). Natural landscapes generally demonstrate a more pronounced stress-reducing effect and a greater potential for attention restoration than artificial hard landscapes (Ma et al., 2026; Twedt et al., 2016). Moreover, semi-natural spaces such as urban parks, owing to their unique combination of accessibility and ecological features, play a crucial role in promoting public health (Wang et al., 2016). Within the urban park system, plant-specific gardens constitute a distinctive type of green space, serving as key ecological hubs that bridge urban ecological development, public leisure, and the enhancement of residents’ physical and mental well-being (Carrus et al., 2017; Mohamed et al., 2022). Consequently, investigating the restorative effects of plan-specific garden landscapes on human health is of particular importance.

Recent research on health-restorative green spaces has increasingly focused on small-scale urban settings, diverse subjects, and varied methodologies. Studies have confirmed the mental health benefits of cultural ecosystem services in high-density residential environments for the elderly (Kabisch et al., 2021; Liu Y. et al., 2022; Chang et al., 2024; Guo et al., 2023), informing age-friendly design. Other work has compared perceptual dimensions (Yakinlar and Akpinar, 2022), landscape types (Wang et al., 2020), vegetation structures (Duan et al., 2024; Hao et al., 2024), and canopy characteristics (Lin et al., 2023) in relation to stress recovery. Virtual reality has been applied to assess preferences and psychological effects of park pavilions (Luo et al., 2022), while empirical evidence has clarified the roles of auditory, olfactory, and visual perceptions in restorative processes (Korpilo et al., 2024; Li et al., 2025; Qi et al., 2022). Collectively, these studies underscore the growing prominence of restorative environment research, with expanding technical approaches and study populations (Ma et al., 2026; Yang et al., 2023).

Previous research has compared restorative effects of various green landscapes, however, few studies have examined the restoration effects of plant-specific gardens. Compared with comprehensive parks and community green spaces, plant-specific gardens are uniquely superior in biodiversity, seasonal landscape change, and cultural symbolism. Existing studies on these gardens focus on aromatic species, moving beyond traditional display toward therapeutic design. For aromatic gardens, studies have recommended their placement in medical settings and used EEG to quantify stress restoration via auditory-olfactory interactions in campus greens paces (Ding et al., 2025); planning scholars have proposed large-scale and campus-based aromatic gardens based on plant traits for public health (He et al., 2019; Li et al., 2024). Multi-sensory evidence indicates that visually, plant color and morphology modulate perceptual responses (Li et al., 2019), with green plants inducing relaxation and calmness, whereas red and dark green increase energy (Elsadek et al., 2013; Hye et al., 2012; Elsadek et al., 2017); olfactorily, aromatic plants and roses induce physiological relaxation, lower blood pressure, and improve mood (Zhang et al., 2024; Gao and Yao, 2012; Igarashi et al., 2014). Symbolic plants subconsciously facilitate psychological restoration (Wu et al., 2023). Additionally, a peony-viewing program during full bloom promotes relaxation in middle-aged and elderly individuals (Zhao et al., 2019). Empirical understanding of how different landscape elements in plant-specific gardens affect psycho-physiological restoration remains limited. Although previous work has examined vegetation structure (Hao et al., 2024; Lin et al., 2023; Luo et al., 2022; Korpilo et al., 2024), landscape openness (Duan et al., 2024), plant color (Li et al., 2019; Elsadek et al., 2013; Hye et al., 2012; Elsadek et al., 2017), aroma (Zhang et al., 2024; Gao and Yao, 2012; Igarashi et al., 2014), and phenological stage (Zhao et al., 2019). The specialized garden with different topographic characteristics has remained marginalized in restorative environment research. Its mechanisms for regulating physical and mental health through various factors such as terrain differences, spatial layers, plant growth conditions, and cultural characteristics have yet to be systematically explained.

This study takes peony gardens in various regions as examples. As a specialized garden type with long history and rich cultural meaning, they have developed three spatial forms: Flatland peony garden (FPG), Terraced peony garden (TPG), and Mountainous peony garden (MPG) (Chen, 2015). Using the Trier Social Stress Test (TSST), virtual reality (VR), and biofeedback, we examined the psycho-physiological restorative effects of three peony garden types (MPG, TPG, FPG) versus an indoor environment (IE) on university students. Physiological parameters and mood states were assessed across stages. The VR landscapes represented semi-managed or fully managed green spaces rather than primitive or wild, commonly found in specialized gardens. We aimed to elucidate the impacts of peony garden viewing on university students and inform health-promoting peony garden design in suitable tree peony growing regions.

## 2 Materials and methods

### 2.1 The environments

Peony gardens are ornamental landscapes serving floral display, aesthetic appreciation, education, and cultural recreation. With millennia-old aesthetic and cultural values, they represent a distinct typology in urban green spaces, botanical gardens, and cultural tourism zones. Based on terrain, spatial layout, and growth habits, they are classified as mountainous, terraced, or flatland types (Chen, 2015). Twelve peony gardens from different regions were initially selected, including mountainous (Chongqing), terraced (Gansu), and flatland urban examples (Xi’an, Luoyang, Heze) (Figure 1). All panoramas were taken on sunny days and classified by type and scale. Initial screening indicated that these peony gardens effectively conveyed the intrinsic traits and cultural associations of tree peonies, with comparable visual quality and minimal external disturbance, thus serving as suitable comparison scenes. Consequently, the MPG and TPG study sites were chosen from the Dianjiang and Caojiaping gardens, respectively, and the FPG sites from Xi’an, Shenzhou, Caozhou, and Heze Baihua peony gardens. Specific scenes were selected based on Attention Restoration Theory criteria and spatial openness (Wang et al., 2016), and regional flowering phenology was accounted for to ensure consistent and feasible fieldwork.

**Figure 1:**
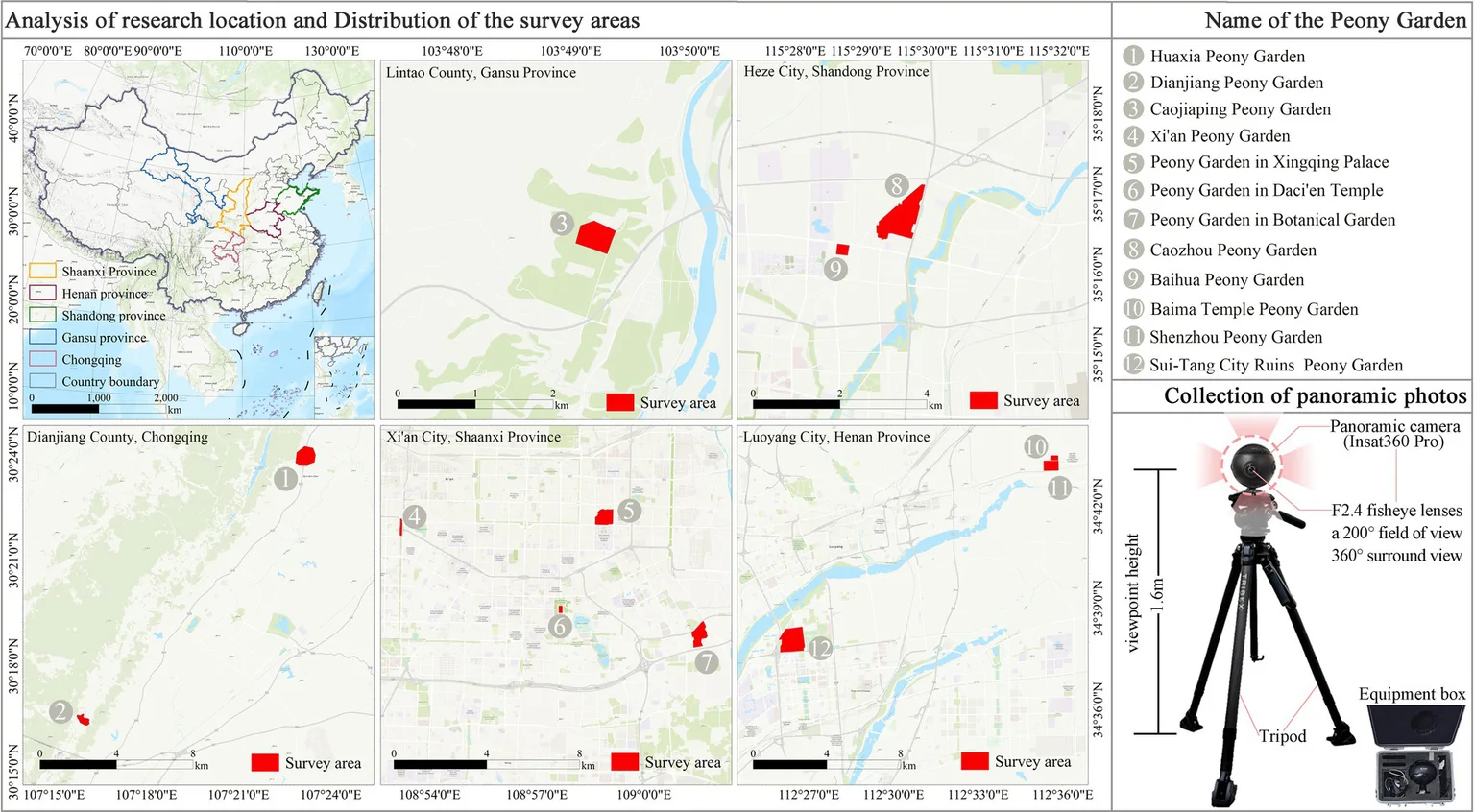
Location of the research and distribution of the survey sites.

Scene production involved three steps. First, from April to May, landscape materials were collected sequentially from the peony gardens using a panoramic camera (Insta360 Pro with 6 F2.4 fisheye lenses, a 200 °field of view, and a spherical 360 °circular layout) at a viewpoint height of 1.6 m (Figure 1) (Wang et al., 2016; Guo et al., 2020). Shooting avoided peak crowds, ensuring no pedestrians, vehicles, or debris appeared in the photos. A total of 54 panoramic photos were taken. Second, the selected photos were further assessed by the garden management staff and researchers from Xi’an Polytechnic University to confirm their representativeness of the key landscape elements (such as spatial hierarchy, plant coverage, visual complexity, etc.) across the three peony garden types (Wang et al., 2016). After discussion, six scenes for each of MPG, TPG, and FPG were selected for the study. All photos had a resolution of 7,680 × 3,840 pixels. IE panoramic photos focused on office space using the same method. Third, Adobe Premiere Pro CS6 software was used to create a 6-min virtual video for each VR landscape (MPG, TPG, FPG, and IE) (Figure 2; Supplementary Table 1). The six scenes were displayed sequentially for one minute each, presented in a random order. Cross-dissolve transitions of 1-s duration were applied between successive scenes in the video. The videos were imported into Pico Goblin A7210 VR glasses (Goertek Inc., China) for trial viewing. All participants viewed identical materials. The six-minute video length was based on previous studies that found physiological and psychological effects in comparable short time frames ranging from six to eight minutes (Wang et al., 2016; Guo et al., 2020). During pre-testing, some subjects began to show impatience at eight minutes, but most were able to watch an six-minute video without becoming noticeably impatient.

**Figure 2:**
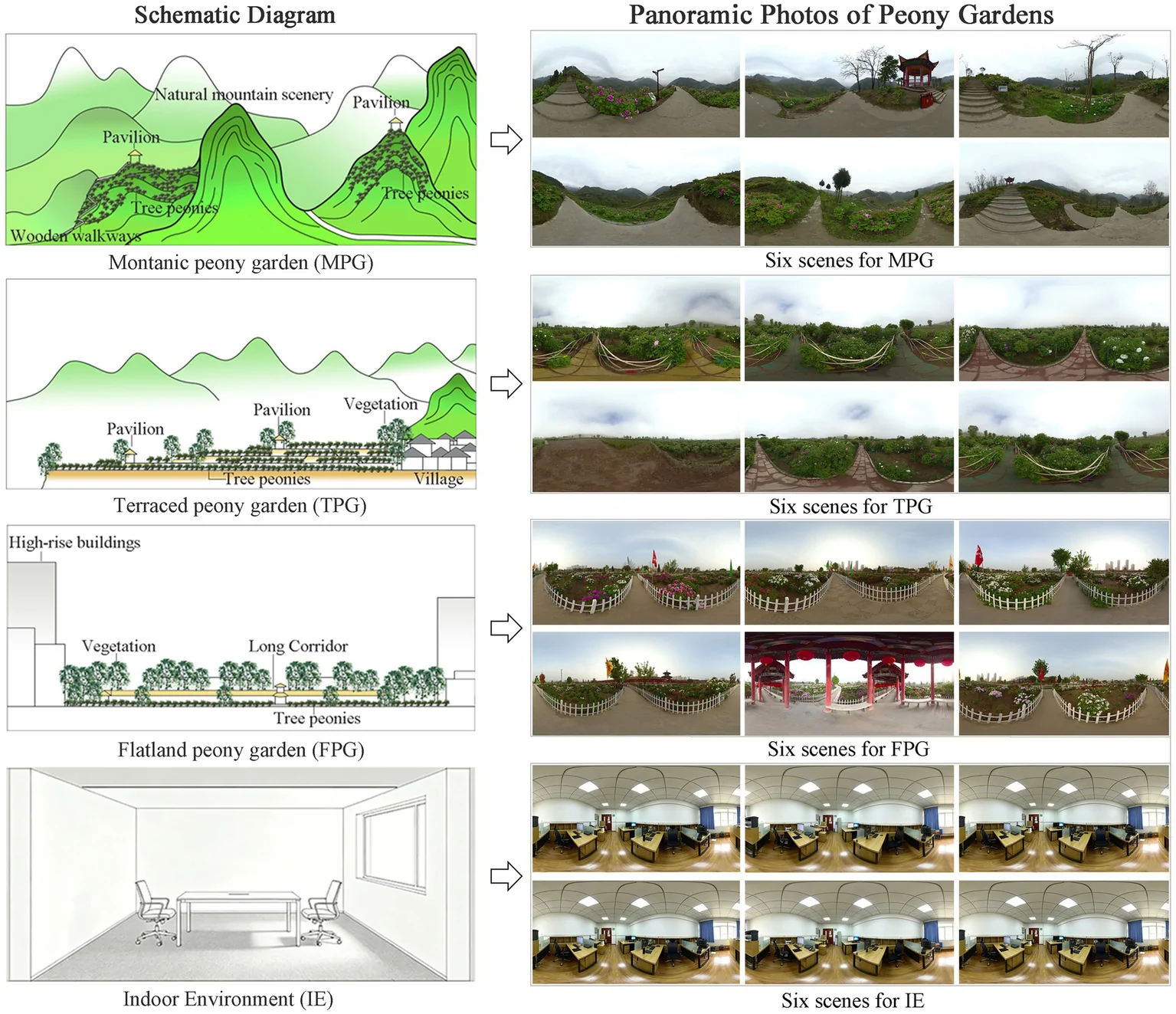
VR scenes of each experimental group.

### 2.2 Participants

A total of 224 volunteer college students aged 20–28 years (mean 23 ± 1.5 years) with normal color vision participated in this study. Exclusion criteria included symptoms of upper respiratory tract infection, neurological or psychiatric disorders, hypertension, cardiovascular diseases, a history of smoking (Frye et al., 1990). Female participants reporting menstruation on the test day were also excluded as a precautionary measure intended to reduce potential variability in test performance associated with menstrual symptoms (e.g., pain, discomfort, or fatigue) and cycle-related physiological changes. However, given the inconsistent evidence regarding menstrual-cycle effects on cognitive function, this criterion was not intended to imply that menstruation itself adversely affects cognitive or perceptual performance (Sundström Poromaa and Gingnell, 2014). To avoid interference with EEG headband measurements, participants refrained from spraying any fragrances or perfumes into their hair within 12 h before the experiment. Subjects were randomly assigned (50% male, 50% female) to watch one of the four VR scenes, with fifty-six subjects watching each virtual video. Prior to the study, each participant received a full explanation and provided written informed consent, with the freedom to withdraw at any time.

### 2.3 Restorative evaluations using virtual reality

Virtual reality (VR) is an effective emotion-inducing medium (Moura et al., 2021) capable of simulating highly realistic environments (Mattila et al., 2020). Unlike conventional two-dimensional media (e.g., photographs), VR provides richer environmental information and fosters immersive experiences rather than passive observation, generating active and passive restorative effects on physical and mental well-being comparable to those of real natural environments (Reese et al., 2021). Cumulative empirical evidence confirms that immersive VR-based natural experiences can effectively promote mental health and alleviate pain and psychological stress (Li et al., 2025; Browning et al., 2019; Wang et al., 2019; Huang et al., 2020; Yu et al., 2020). This supports the use of VR simulations of different peony garden landscapes to evaluate physical and mental restoration in the present study.

### 2.4 Stress induction

The Trier Social Stress Test (TSST) is a well-validated paradigm for inducing psychological stress in controlled indoor settings (Guo et al., 2020; Birkett, 2011; Yang et al., 2011). It comprises two sequential three-minute tasks: an impromptu speech (e.g., a job application self-introduction or a designated topic) and a mental arithmetic task (serial subtraction of 13 from 1,022). Participants must answer quickly and accurately, any error requires restarting from 1,022.

### 2.5 Measures of stress level

#### 2.5.1 Physiological indexes

Electroencephalogram (EEG) signals were recorded from the Fp1 position using a portable Mind Wave-EEG headset (NeuroSky; Beijing Vision Technology Co., Ltd., Beijing, China). The headset comprises a headband, an ear-clip, a sensor arm with the EEG electrode, and a Bluetooth USB. It measures raw signals, power spectrum (delta, theta, low alpha, high alpha, low beta, high beta, low gamma, high gamma), and levels of mediation and attention. Previous studies have used this device to examine relaxation effects in older adults via low/high alpha and low/high beta indices (Ahmad et al., 2017) and restoration effects of forest walking in young adults using high alpha and beta indices (Hassan et al., 2018). Alpha and beta waves have also been employed to record physiological stress responses (Kalas and Momin, 2016; Al-Shargie et al., 2015). Accordingly, low alpha, high alpha, low beta, and high beta were selected as evaluation indicators in this experiment.

Heart rate and heart rate variability (HRV) were measured using a wearable electrocardiogram system (Polar V800; Polar Electro, Finland) to quantify autonomic nervous system responses. The raw recording durations varied across experimental phases: 4 min for baseline, 6 min for stress, and 6 min for recovery. To eliminate potential confounding effects of unequal recording durations on frequency-domain HRV comparisons, the final 4-min continuous R-R interval segment from each phase was extracted for spectral analysis. Frequency spectra were generated with HRV analysis software (University of Vigo, Spain) (Rodríguez-Liñares et al., 2014). Power levels of low frequency (LF, 0.04–0.15 Hz) and high frequency (HF, 0.15–0.40 Hz) components were calculated using the maximum entropy method from continuous R-R intervals. Both LF and HF were expressed as absolute power (unit: ms^2^) instead of normalized units. HF reflects parasympathetic nervous activity, and the LF/HF ratio estimates sympathetic nerve activity (Malik, 1996).

#### 2.5.2 Psychological indexes

Psychological testing was used to assess participants’ mental states via standardized rating scales. The validated Chinese version of the State–Trait Anxiety Inventory-State subscale (STAI-S) was adopted (Li and Qian, 1995), which comprises 20 items, each scored 1–4; total state-anxiety scores range from 20 to 80, with higher scores indicating greater anxiety. Its Cronbach’s *α* ranging from 0.83 to 0.92 among Chinese college students. The validated Chinese version of the Profile of Mood States (POMS) was also employed (Zhu, 1995). It includes includes 35 items across six dimensions: anger-hostility (A-H), depression-dejection (D-D), tension-anxiety (T-A), confusion-bewilderment (C-B), vigor-activity (V-A), and fatigue-inertia (F-I). Higher dimension scores reflect higher degrees of the corresponding mood state, with subscale Cronbach’s *α* ranging from 0.71 to 0.89 in Chinese college-student samples (Wang et al., 2020; Zhao et al., 2019).

The Perceived Restorativeness Scale (PRS) was used to assess subjective recovery, with Cronbach’s *α* of >0.769–0.936 (Ye et al., 2010). It comprises 23 items across four dimensions: Being Away (6 items), Fascination (7 items), Coherence (4 items), and Compatibility (6 items) (Browning et al., 2019). Example items include “This place is a refuge from unwanted distractions” (Being Away), “This place is fascinating” (Fascination), “Here is a clear order in the physical arrangement of the place” (Coherence), and “This place does not place demands on me to act in a way I would not choose” (Compatibility). Items are rated on a 5-point scale from −2 to +2, higher scores indicate a greater contribution of that dimension to landscape restoration. The scores of items that indicated negative experiences were reversed before conducting the analysis. Each subscale score was computed as the sum of its corresponding items. The overall PRS score was calculated as the mean of the four subscale scores. Higher subscale and overall PRS scores indicate greater perceived restorativeness of the landscape environment.

#### 2.5.3 The procedure

This experiment included four groups: IE, FPG, TPG, and MPG. All procedures were conducted in a quiet indoor space away from the teaching area, with controlled temperature (23 ± 2 °C) and relative humidity (40–50%). The protocol comprised three standardized stages. Preparation phase: Participants received an experimental briefing and were equipped with heart rate sensors and EEG devices. Measurement phase: This included baseline, stress, and recovery stages. During the 4-min baseline, participants sat relaxed while physiological indicators were recorded. In the stress stage, participants completed the 6-min TSST (impromptu speech and mental arithmetic), followed by POMS and STAI questionnaires. In the recovery stage, participants wore VR headsets and viewed one of the experimental landscape scenes for 6 min (Guo et al., 2020), physiological indices were recorded, followed by POMS, STAI, and PRS. Concluding phase: All data were collected and archived, and participants removed devices before exiting orderly (Figure 3).

**Figure 3:**
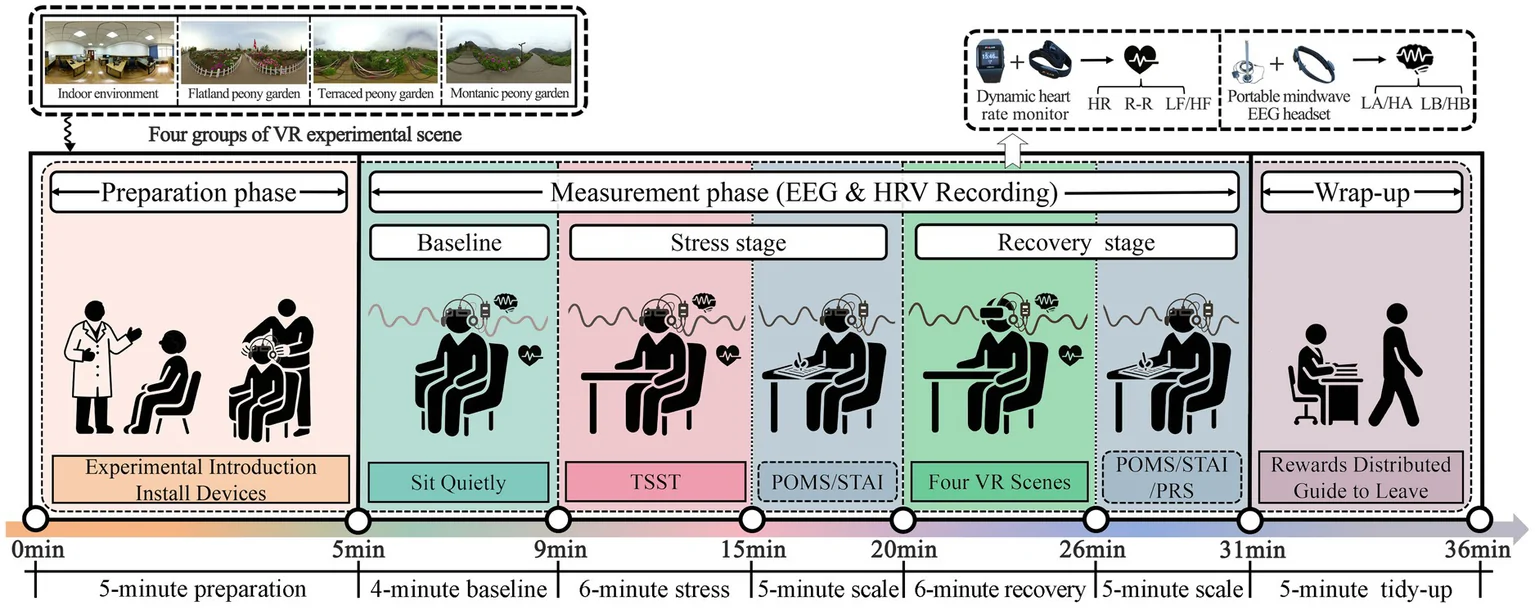
Experimental content and procedure.

### 2.6 Data and statistical analysis

Data were analyzed using SPSS 19.0 (IBM® SPSS® Statistics, Armonk, NY). A paired sample *t*-test was used to compare the means for psycho-physiological data between the stress and the recovery stages. The psycho-physiological changes among the different peony garden scenes were determined by one-way analysis of variance (ANOVA). Partial eta squared (*η_p_*^2^), indicating that the independent variables can explain the size of the overall variance of the dependent variables, was used to report the estimate of effect sized for the ANOVA. In the ANOVA analyses, LSD, S-N-S, and Bonferroni corrections were applied for multiple comparisons. Internal reliability of the POMS, STAI-S, and PRS subscales was analyzed with Cronbach’s *α*. Pearson correlation analysis examined relationships between psychological indicators and PRS overall scores. The data were expressed as the mean ± standard deviation (mean ± SD). In all comparisons, a *p*-value of < 0.05 was considered statistically significant. Effect size was reported using Cohen’ s *d*, representing the standardized difference between the two sets of means for A paired sample *t*-test.

## 3 Results

### 3.1 Tests for the baseline levels and the validity of the stressor

Prior to comparing stress and recovery stages, baseline values were analyzed. One-way ANOVA revealed no significant group differences in HR (*p* = 0.861, *η*_p_^2^ = 0.005), R-R intervals (*p* = 0.769, *η*_p_^2^ = 0.008), Low Alpha (*p* = 0.900, *η*_p_^2^ = 0.004), High Alpha (*p* = 0.712, *η*_p_^2^ = 0.010), Low Beta (*p* = 0.876, *η*_p_^2^ = 0.005), and High Beta (*p* = 0.526, *η*_p_^2^ = 0.016). The absence of significant baseline differences across groups ruled out initial physiological heterogeneity as a potential confounder in interpreting intervention-induced changes.

We conducted paired *t*-tests to examine the mean values of physiological data from the baseline to the time immediately following the TSST for all participants. The TSST provoked a significant decrease in R-R intervals (*p* = 0.001, Cohen’s *d* = 1.04), HF (*p* < 0.01, *d* = 0.14), the low alpha mean values (*p* < 0.01, *d* = 0.84) and the high alpha mean values (*p* = 0.003,*d* = 0.76). There was a significant increase in HR (*p* < 0.01, *d* = 1.53), LF/HF (*p* < 0.01, *d* = 0.29), the low beta mean values (*p* < 0.01, *d* = 0.87) and the high beta mean values (*p* < 0.01, *d* = 0.85) (Supplementary Table 2). These data indicated that the TSST produced effective stress stimulation for subjects.

### 3.2 Tests for physiological effects of different scenes

#### 3.2.1 HR, R-R interval, HF, LF/HF

Paired *t*-tests revealed significant differences in HR, R-R intervals, LF/HF, and HF from the stress stages to the recovery stages. Except for the IE, viewing peony garden landscapes significantly altered HR (*p* = 0.000, FPG: *p* = 0.000, *d* = 0.70; TPG: *p* = 0.000, *d* = 0.67; MPG: *p* = 0.000, *d* = 1.11), R-R intervals (FPG: *p* = 0.010, *d* = 0.35; TPG: *p* = 0.000, *d* = 0.74; MPG: *p* = 0.000, *d* = 0.75), LF/HF (FPG: *p* = 0.034, *d* = 0.49; TPG: *p* = 0.000, *d* = 1.86; MPG: *p* = 0.000, *d* = 2.22) and HF values (FPG: *p* = 0.013, *d* = 0.57; TPG: *p* = 0.009, *d* = 0.63; MPG: *p* = 0.000, *d* = 0.96) (Supplementary Table 3; Figure 4).

**Figure 4:**
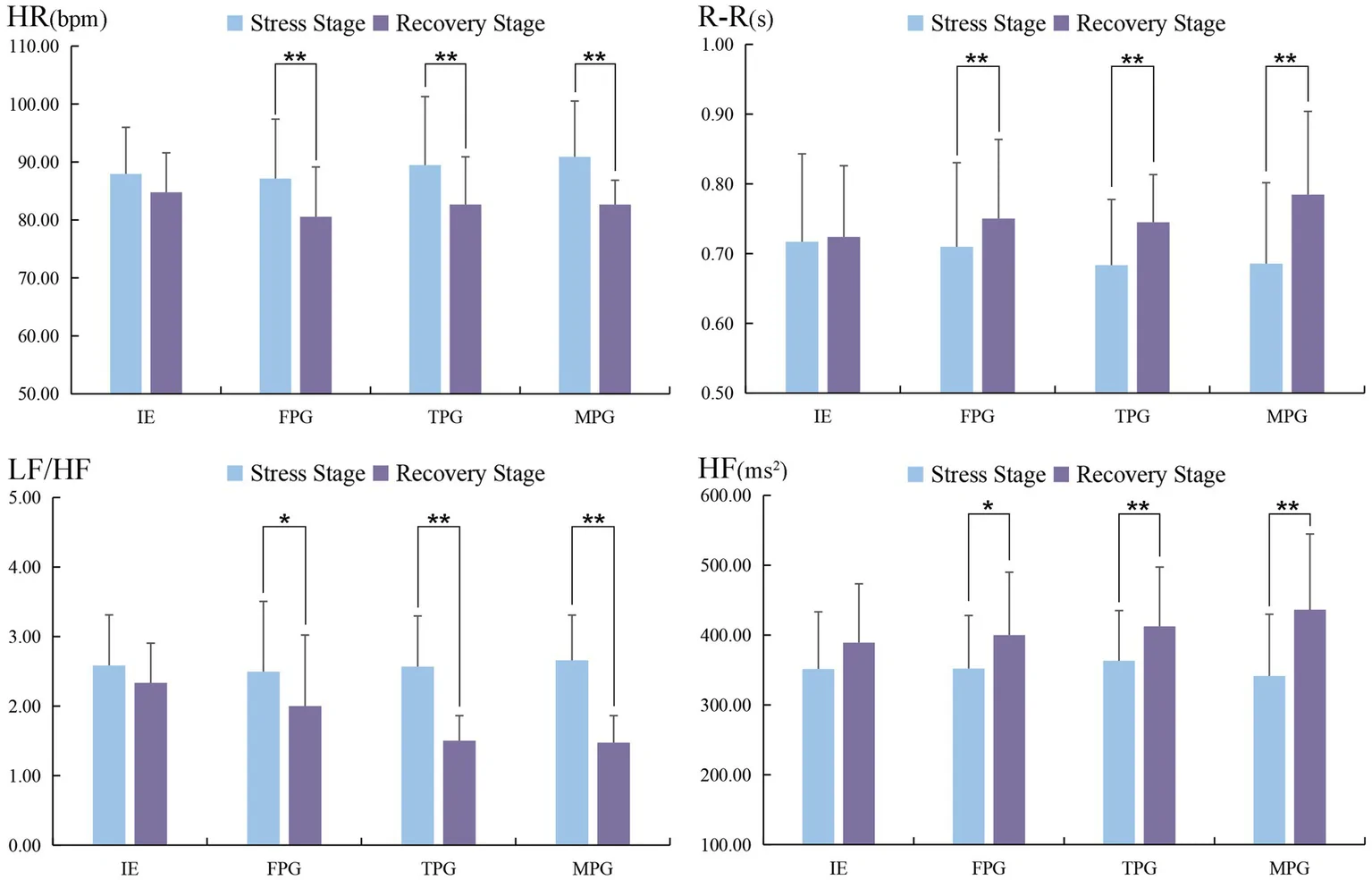
Comparison of mean values of heart rates, R-R intervals, LF/HF and HF from the stress stages for the recovery stages. IE, Indoor environment; FPG, Flatland peony garden; TPG, Terraced peony garden; MPG, Mountainous peony garden. *n* = 56, mean ± SD, *p < 0.05, **p < 0.01, paired-sample *t*-test.

One-way ANOVA revealed that the short-term viewing program induced changes in R-R intervals (*p* = 0.006, *η_p_*^2^ = 0.064) and LF/HF (*p* = 0.000, *η_p_*^2^ = 0.126) across different peony garden landscapes. No significant R-R interval changes were found among IE, FPG, and TPG. LF/HF changes between IE and FPG were not significant. HR (*p* = 0.012, *η_p_*^2^ = 0.027) and HF (*p* = 0.047, *η_p_*^2^ = 0.012) changes were significant only between IE and MPG, and changes among IE, FPG, and TPG were not significant (Figure 5).

**Figure 5:**
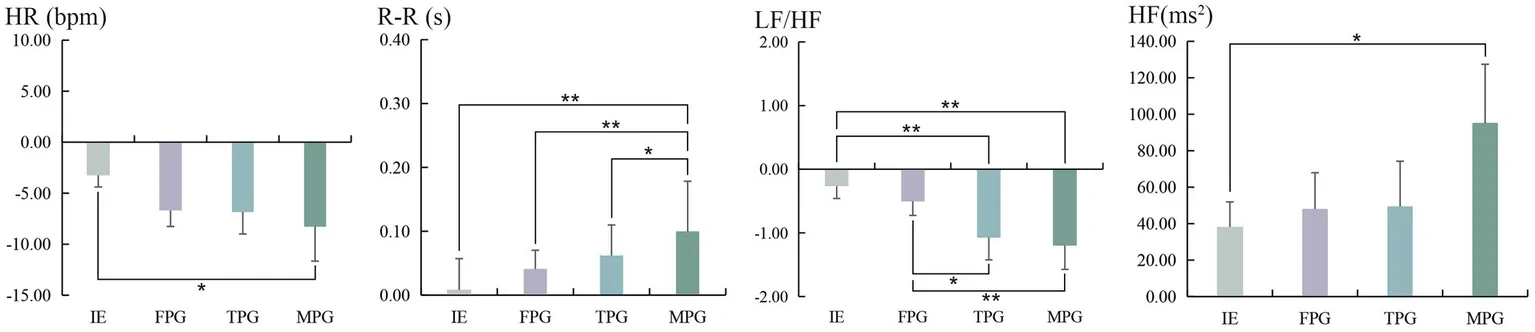
Comparison of physiological index changed values among the different peony garden scenes at the recovery stages. IE, Indoor environment; FPG, Flatland peony garden; TPG, Terraced peony garden; MPG, Mountainous peony garden. *n* = 56. mean ± SD. *p < 0.05, **p < 0.01, one-way ANOVA.

#### 3.2.2 EEG

The mean values of low and high alpha increased during the recovery stages, comparing to the stress stages. Paired *t*-tests revealed that low alpha mean values of viewing peony gardens MPG increased the most (*p* = 0.000, *d* = 1.03), TPG took second place (*p* = 0.000, *d* = 0.90), and FPG changed relatively little (*p* = 0.007, *d* = 0.69). The high alpha mean values exhibited a similar trend of increase in four scenes (FPG: *p* = 0.000, *d* = 1.00; TPG: *p* = 0.000, *d* = 1.13; MPG: *p* = 0.002, *d* = 1.14). Moreover, the low beta mean values showed significant differences while viewing FPG (*p* = 0.046, *d* = 0.48), TPG (*p* = 0.040, *d* = 0.56), and MPG (*p* = 0.033, *d* = 0.50). The high beta mean values also significantly decreased in the different scenes (FPG: *p* = 0.049, *d* = 0.42; TPG: *p* = 0.022, *d* = 0.57; MPG: *p* = 0.014, *d* = 0.60) (Supplementary Table 3; Figure 6). There was no significant difference observed in the indoor environment (IE).

**Figure 6:**
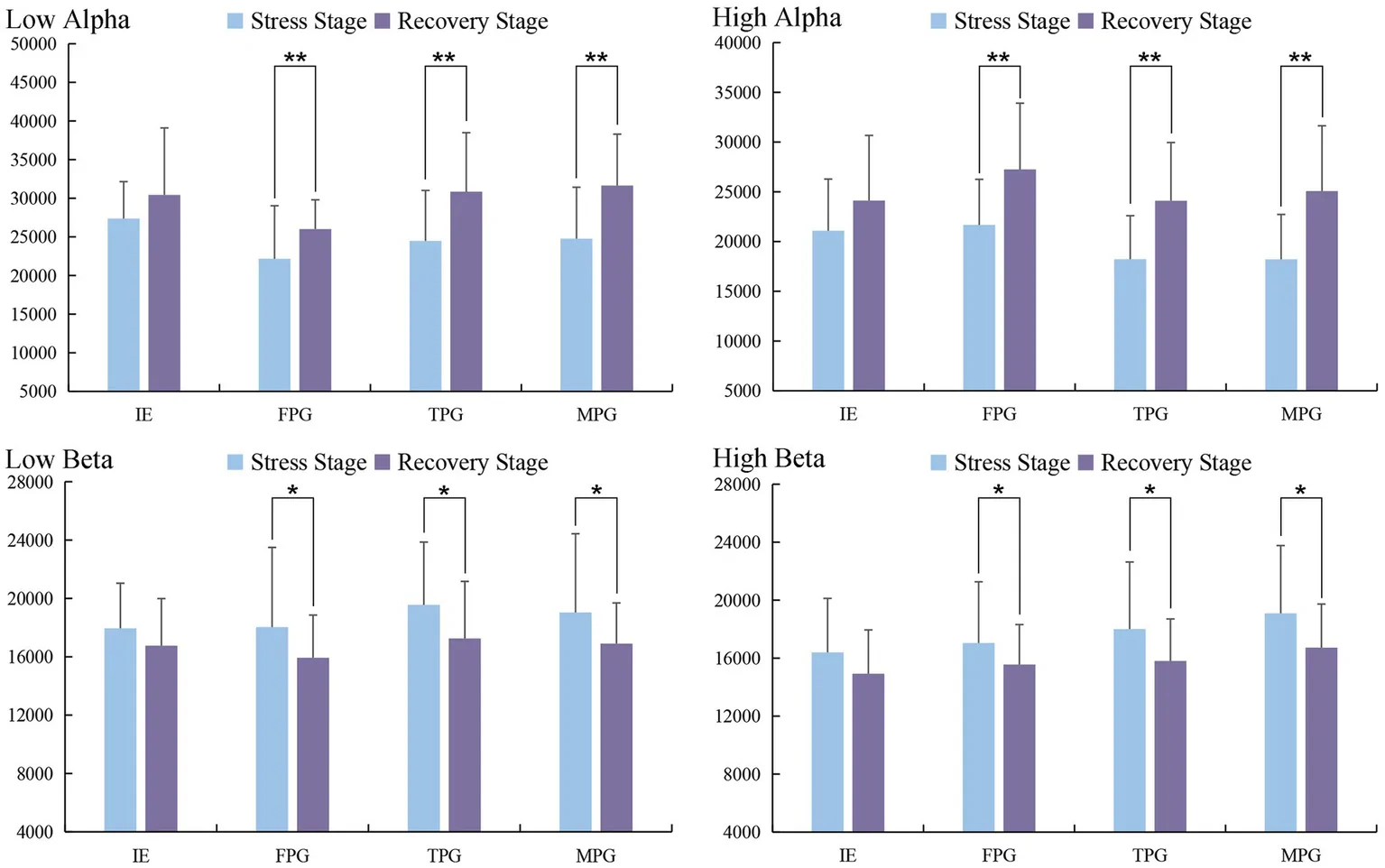
Comparison of mean values of low alpha, high alpha, low beta, and high beta brainwaves from the stress stages for the recovery stages. IE, Indoor environment; FPG, Flatland peony garden; TPG, Terraced peony garden; MPG, Mountainous peony garden. *n* = 56, mean ± SD, *p < 0.05, **p < 0.01, paired-sample *t*-test.

One-way ANOVA revealed that the short-term viewing program induced changes in low alpha (*p* = 0.000, *η_p_*^2^ = 0.637) and high alpha (*p* = 0.000, *η_p_*^2^ = 0.607) across different peony garden landscapes. No significant difference was found in low alpha between TPG and MPG, or in high alpha between TPG and FPG. Changes in low beta (*p* = 0.837, *η_p_*^2^ = 0.006) and high beta (*p* = 0.824, *η_p_*^2^ = 0.015) were not significant among IE, FPG, TPG, and MPG (Figure 7).

**Figure 7:**
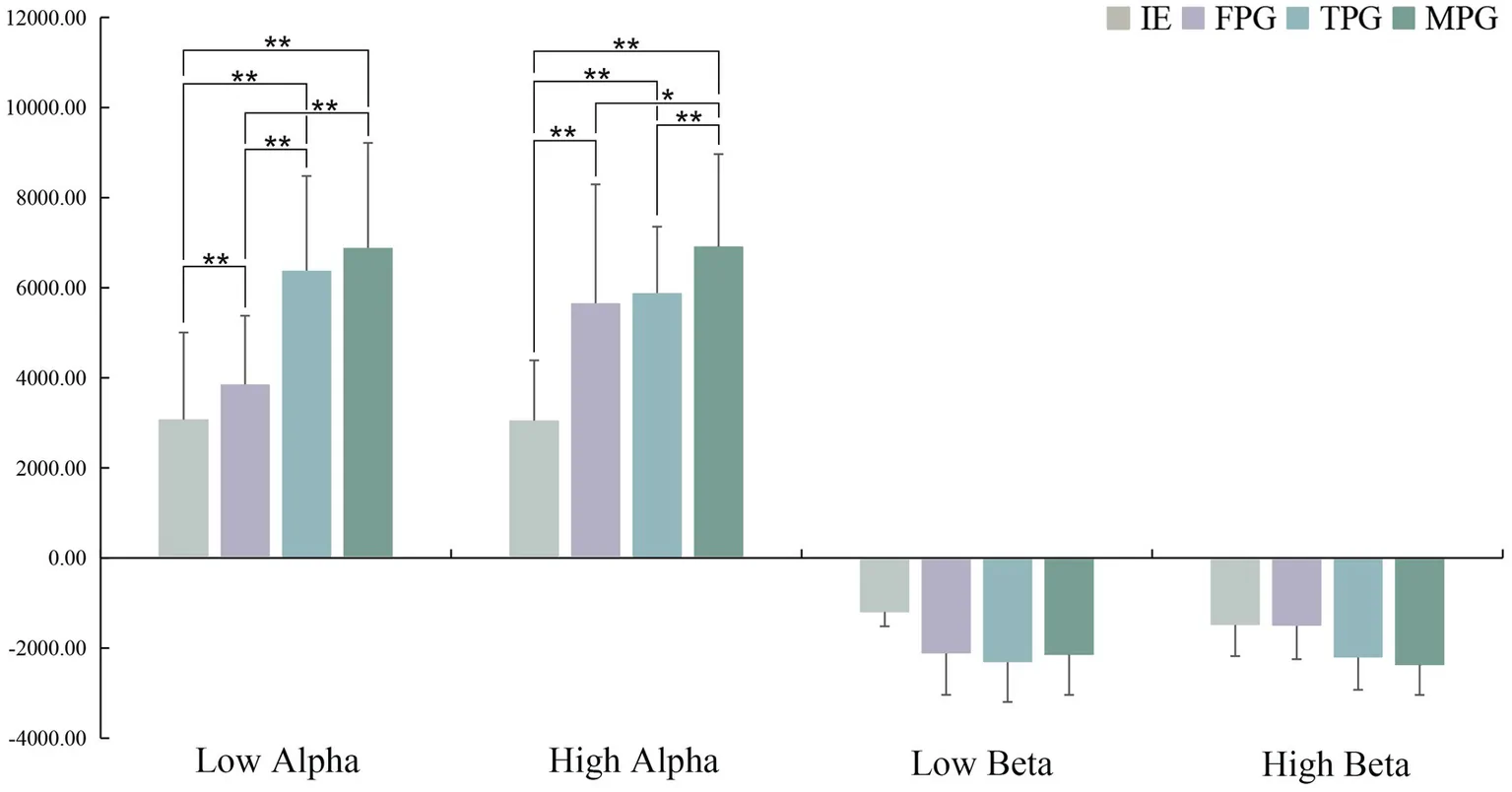
Comparison of EEG wave changed values among the different types of peony garden landscapes. IE, Indoor environment; FPG, Flatland peony garden; TPG, Terraced peony garden; MPG, Mountainous peony garden. *n* = 56. mean ± SD. *p < 0.05, **p < 0.01, one-way ANOVA.

### 3.3 Tests for psychological effects of different scenes

#### 3.3.1 State anxiety

Results of the STAI-S showed that most subscales had good reliability among participants in this study, with Cronbach’s *α* ranging from 0.842 to 0.965. Paired *t*-tests showed that the STAI-S scores significantly decreased after viewing the peony garden scenes (for all *p* < 0.01, *d* = 1.18–1.76) (Supplementary Table 4; Figure 8A). No significant difference was found in levels of anxiety relief in the indoor environment. One-way ANOVA indicated that viewing different peony garden landscapes significantly altered anxiety (*p* = 0.000, *η_p_*^2^ = 0.249). There was no significant differences between FPG and TPG (Figure 8B). The change in STAI-S from the stress stage to the recovery stage was largest in the MPG (15.86), followed by TPG (12.75), and FPG (11.72).

**Figure 8:**
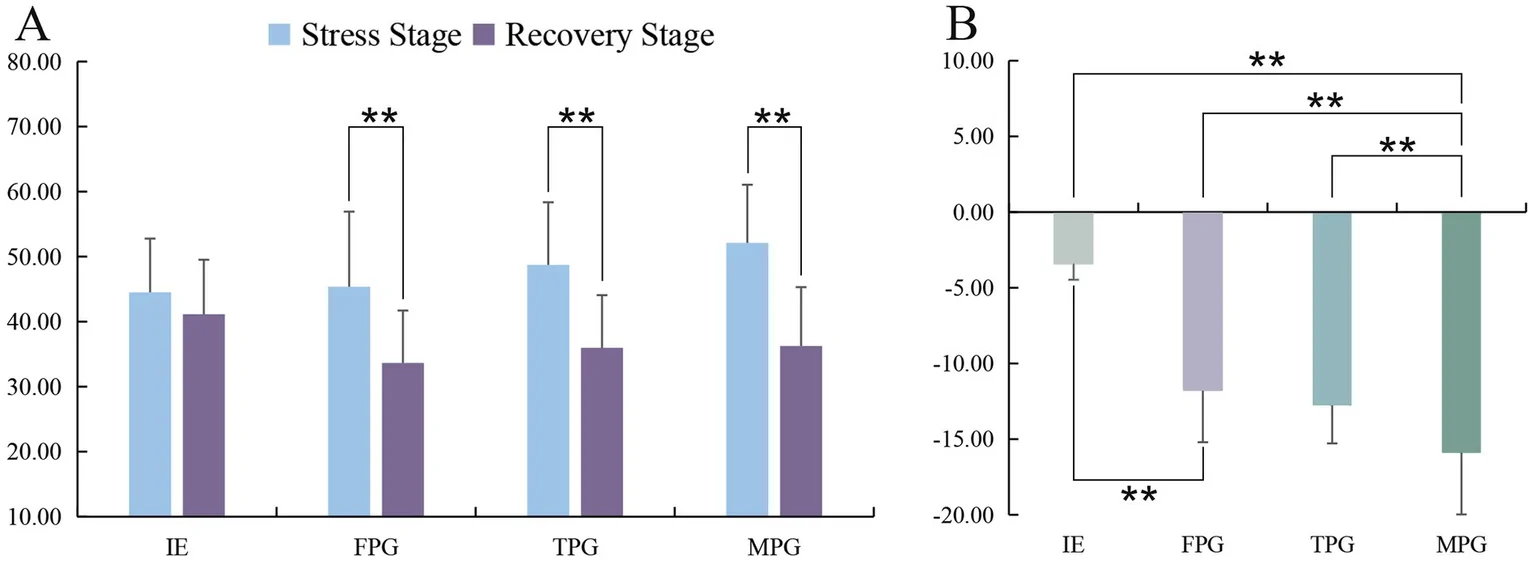
STAI-S scores of the subjects in different experimental groups. **(A)** Changes during the stress stages and recovery stages. *n* = 56, mean ± SD, **p < 0.01, paired-sample *t*-test. **(B)** Comparison of STAI-S change scores among the different scenes. IE, Indoor environment; FPG, Flatland peony garden; TPG, Terraced peony garden; MPG, Mountainous peony garden. *n* = 56, mean ± SD, **p < 0.01, one-way ANOVA.

#### 3.3.2 Mood states

The POMS subscales showed good reliability in this study, with Cronbach’s α ranging from 0.723 to 0.891. Compared to the stress stages, participants exhibited a significant improvement in mental state after viewing the diverse peony garden scenes. Paired *t*-tests showed significant reductions from the stress stages to the recovery stages in five negative POMS subscales: T-A (*p* < 0.01, *d* = 0.85–1.47), A-H (*p* < 0.01, *d* = 0.76–0.81), F-I (*p* < 0.01, *d* = 0.36–1.04), D-D (*p* < 0.01, *d* = 0.46–1.45), and C-B (*p* < 0.01, *d* = 0.81–1.81), while positive mood V-A significantly increased (*p* < 0.01, *d* = 0.23–0.70) (Supplementary Table 4; Figure 9). One-way ANOVA indicated that viewing different peony garden landscapes significantly altered T-A (*p* = 0.000, *η_p_*^2^ = 0.275), A-H (*p* = 0.009, *η_p_*^2^ = 0.060), F-I (*p* = 0.000, *η_p_*^2^ = 0.156), D-D (*p* = 0.002, *η_p_*^2^ = 0.081), and C-B (*p* = 0.000, *η_p_*^2^ = 0.258), and positively affected V-A (*p* = 0.004, *η_p_*^2^ = 0.069). Changes in A-H, D-D, and V-A showed no significant differences between IE and FPG, nor between TPG and MPG. Changes in F-I (TPG vs. MPG) and C-B (FPG vs. TPG) were also not significant (Figure 10).

**Figure 9:**
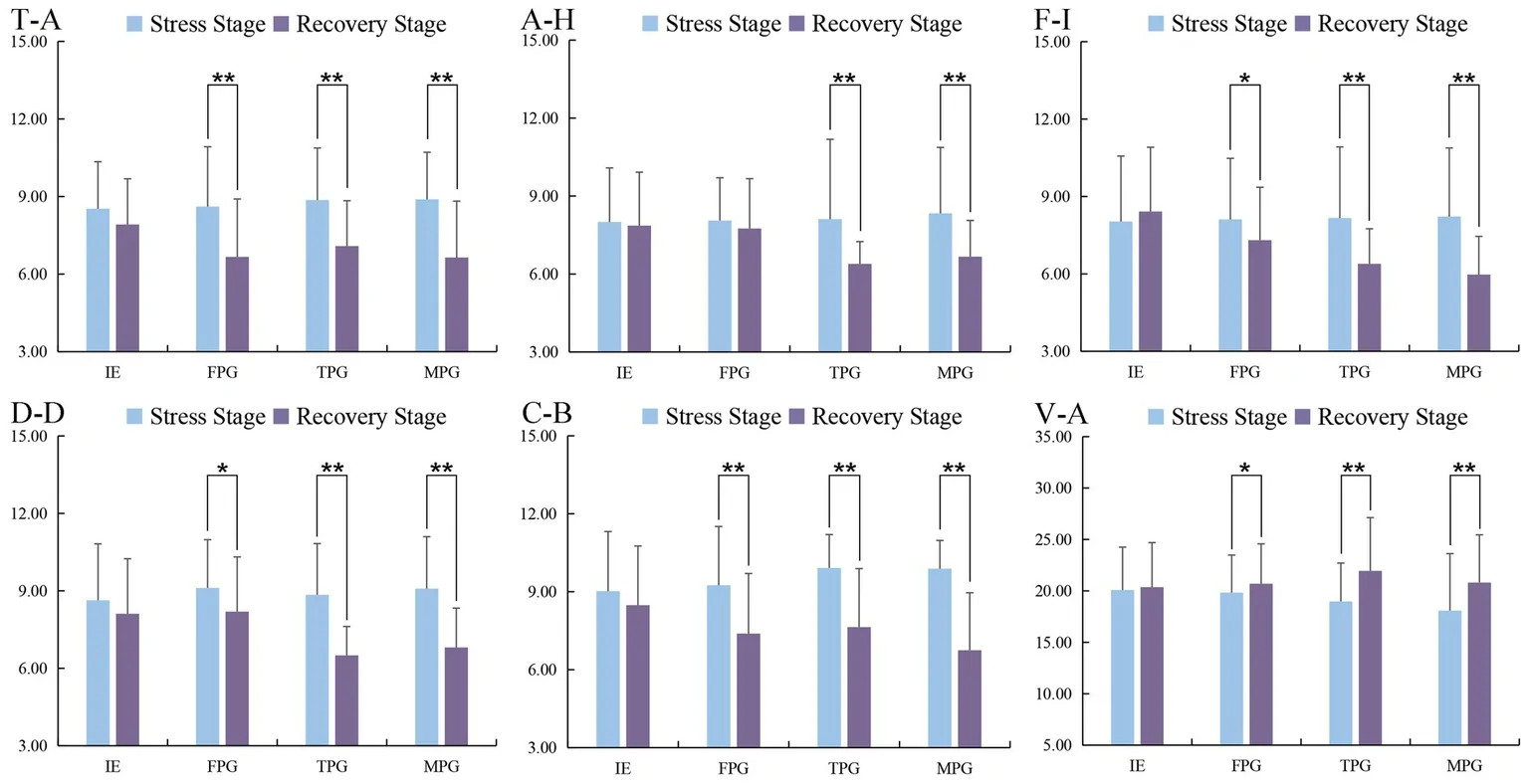
Comparison of mean values of POMS scores between the stress stages and the recovery stages. T-A, tension-anxiety; A-H, anger-hostility; F-I, fatigue-inertia; C-B, confusion-bewilderment; D-D, depression-dejection; V-A, vigor-activity; IE, Indoor environment; FPG, Flatland peony garden; TPG, Terraced peony garden; MPG, Mountainous peony garden. *n* = 56, mean ± SD, *p < 0.05, **p < 0.01, paired-sample *t*-test.

**Figure 10:**
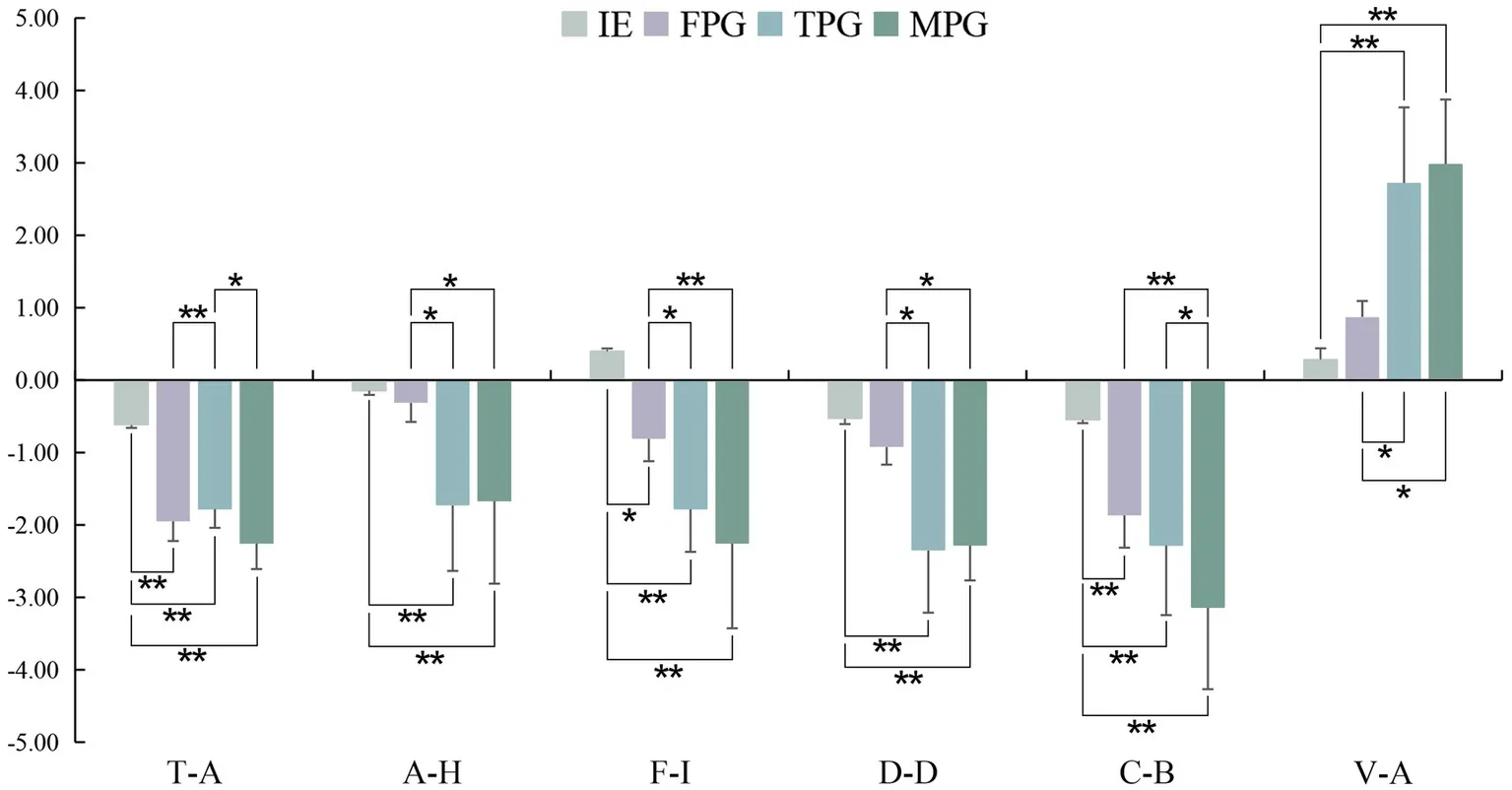
Comparison of changed values in POMS scores among the different peony garden scenes at the recovery stages. T-A, tension-anxiety; A-H, anger-hostility; F-I, fatigue-inertia; C-B, confusion-bewilderment; D-D, depression-dejection; V-A, vigor-activity; IE, Indoor environment; FPG, Flatland peony garden; TPG, Terraced peony garden; MPG, Mountainous peony garden. *n* = 56, mean ± SD, *p < 0.05, **p < 0.01, one-way ANOVA.

### 3.4 Assessing the perceived restorativeness of different scenes

#### 3.4.1 Overall PRS

Reliability analyses showed that the majority of PRS subscales achieved good internal consistency, with Cronbach’s α values between 0.875 and 0.932. The overall perceived restorativeness results showed significant differences among the four scenes (*p* < 0.001, *η_p_*^2^ = 0.151). A Tukey post-hoc test revealed that all three peony garden scenes were perceived as more restorative than the indoor environment (*p* < 0.001). Of the peony garden scenes, The MPG (5.86 ± 1.81, *p* = 0.000), TPG (4.05 ± 2.64, *p* = 0 0.001) and FPG (4.13 ± 2.43, *p* = 0.000) scenes yielded higher perceived restorativeness (PRS) compared with the IE (3.24 ± 1.32), indicating stronger subjectively perceived restorative potential.

Correlation analysis examined relationships between overall PRS and psychological indicators during the recovery stages. PRS scores were negatively correlated with T-A (TPG: *r* = −0.393, *p* = 0.018), A-H (MPG: *r* = −0.385, *p* = 0.021), F-I, D-D, C-B, and anxiety levels (TPG: *r* = −0.436, *p* = 0.008), while positively correlated with V-A (FPG: *r* = 0.598, *p* = 0.000; TPG: *r* = 0.378, *p* = 0.023; MPG: *r* = 0.454, *p* = 0.005) (Figure 11), demonstrating the subjects’ perceived restorativeness after viewing the peony garden scenes was positively correlated with the improvement of psychological emotions.

**Figure 11:**
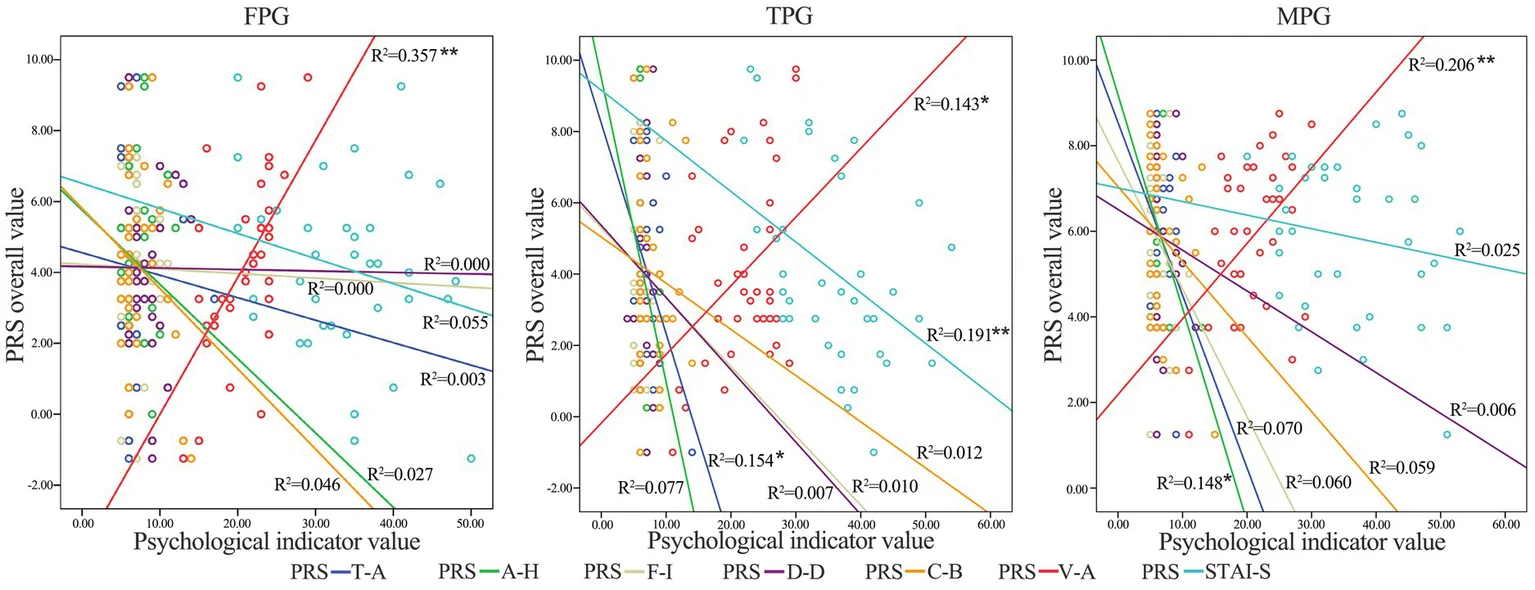
Correlation analysis of psychological indicators and PRS overall scores at the recovery stages. FPG, Flatland peony garden; TPG, Terraced peony garden; MPG, Mountainous peony garden; T-A, tension-anxiety; A-H, anger-hostility; F-I, fatigue-inertia; C-B, confusion-bewilderment; D-D, depression-dejection; V-A, vigor-activity; STAI, anxiety levels. *n* = 56, *p < 0.05, **p < 0.01, correlation analysis.

#### 3.4.2 Subscale scores

To detect the different characteristics of peony garden scenes in stress restoration, scores at the subscale level were calculated and analyzed (Figure 12). For the subscale “being away” (*p* = 0.000, *η_p_*^2^ = 0.208), the scene of MPG was significantly higher than TPG (*p* = 0.008) or FPG (*p* = 0.005). For the same subscale, TPG (*p* = 0.000) and FPG (*p* = 0.001) were rated significantly higher than IE. For the subscale “fascination” (*p* = 0.001, *η_p_*^2^ = 0.094), the scenes of MPG had a higher perceived restorativeness. For the same subscale, TPG (*p* = 0.000) and FPG (*p* = 0.006) was higher rated than IE. For the subscale “coherence” (*p* = 0.010, *η_p_*^2^ = 0.057), MPG, TPG, and FPG were rated higher than IE (*p* < 0.01), but no significant difference was found among MPG, TPG, and FPG. For the subscale “compatibility”(*p* = 0.140, *η_p_*^2^ = 0.017), the scene of MPG and FPG were rated higher than TPG, but no significant difference was found during the all peony garden scenes.

**Figure 12:**
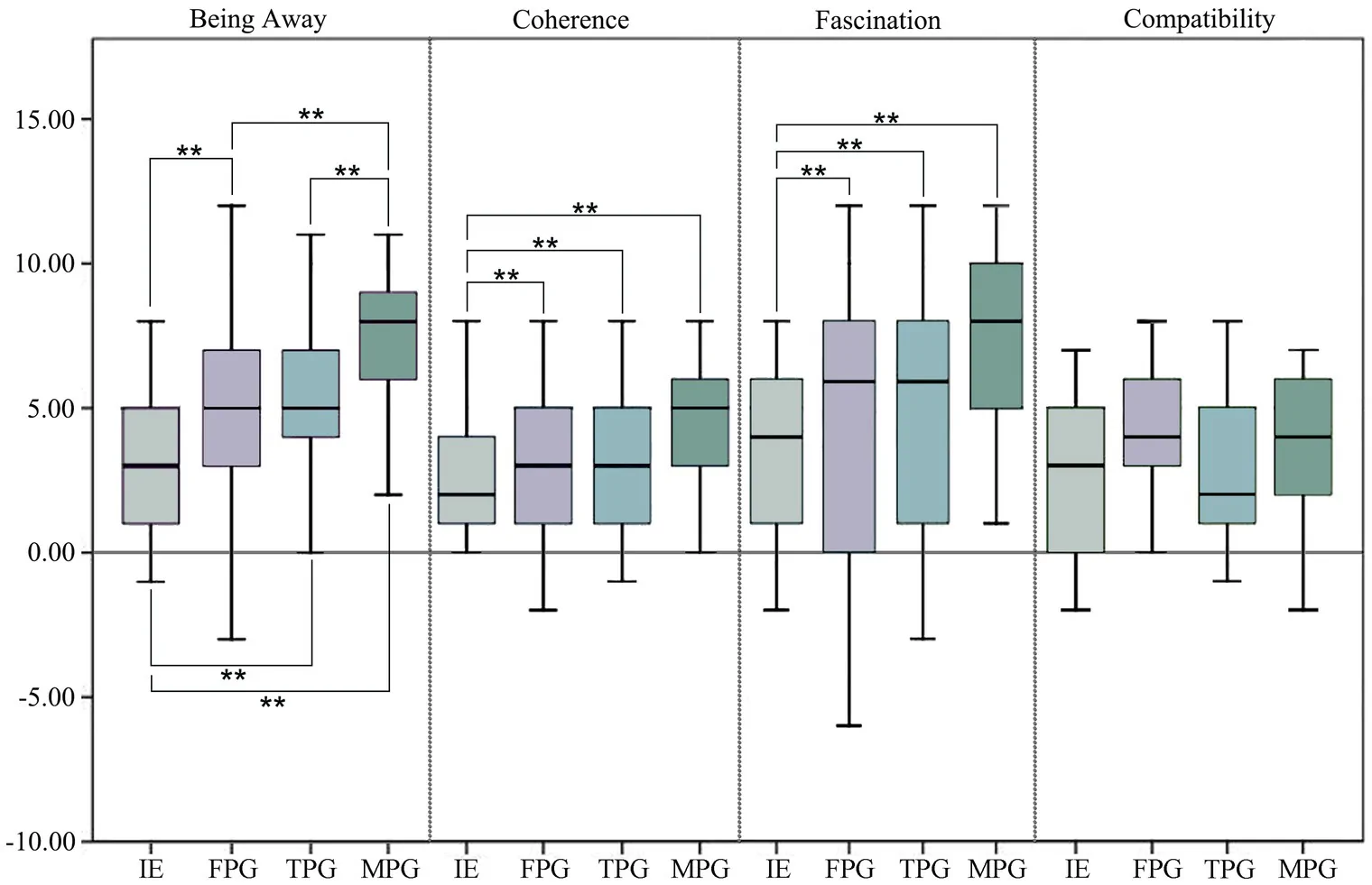
Comparison of the PRS subscale scores among the different peony garden scenes. IE, Indoor environment; FPG, Flatland peony garden; TPG, Terraced peony garden; MPG, Mountainous peony garden. *n* = 56, mean ± SD, **p < 0.01, exploratory analysis.

## 4 Discussion

This study combined the Trier Social Stress Test (TSST) with virtual reality (VR) scene stimulation to examine the restorative effects of peony garden landscapes on stress in college students. During the stress stage, the TSST induced significant increases in HR, LF/HF, and low-and high-beta power, alongside decreases in R-R interval, HF, and low-and high-alpha power, confirming a state of psychological tension and validating the TSST as an effective acute stress paradigm (Yang et al., 2011; Ahmad et al., 2017). In the recovery stage, exposure to VR-simulated peony gardens (vs. the indoor control) produced opposite physiological changes, reductions in HR, LF/HF, and beta, and increases in R-R interval, HF, and alpha, suppressed sympathetic and enhanced parasympathetic nerve activity, thus facilitating relaxation (Malik, 1996). Moreover, peony garden viewing significantly improved emotional states and reduced anxiety, as reflected by decreases in negative subscales (T-A, A-H, F-I, D-D, C-B) and a marked increase in vigor-activity (V-A). Consistent with previous studies (Lin et al., 2023; Qi et al., 2022; Ahmad et al., 2017; Tsunetsugu et al., 2013), peony garden visual stimulation (vs. indoor control) effectively alleviated stress, improved mood, and enhanced positive emotions in college students.

Viewing peony garden scenes significantly facilitated perceived restorativeness in college students. Significant differences among landscape types emerged for the “being away,” “coherence,” and “fascination” subscales of the PRS, but not for “compatibility.” The MPG showed the stronger restorative potential, particularly in evoking “being away” (detachment from routine via environmental contrast) and “fascination” (immersion and curiosity stimulation) (Hao et al., 2024). Furthermore, overall PRS scores were significantly linked to better emotional states. The MPG, TPG, and FPG scenes were perceived as more restorative than the IE, indicating nature-based components promoted perceived recovery better than hardscape components (Hao et al., 2024; Tang and Brown, 2006).

In terms of physiological and psychological changes, MPG triggered relatively larger recovery and relaxation responses across multiple but not all indicators when compared with TPG and FPG. Consistent with previous studies (Tang and Brown, 2006; Ulrich et al., 1991; Liu and Xu, 2016), landscape naturalness and greenness were positively associated with restorative outcomes, with higher naturalness generally facilitating physical and mental recovery. Within this framework, the superior restorative responses observed for MPG in the present study may be attributable to its distinctive topographic and visual characteristics. Specifically, the undulating terrain of MPG generates multi-layered, three-dimensional visual perceptions of blooming peonies. Alternating slopes, valleys, and ridges create dynamic visual interfaces with variable openness and enclosure, while topography-stratified peony distribution, undulating forest edges, and distant mountain silhouettes collectively produce multi-depth visual planes. These spatial features likely enhance landscape diversity and visual dynamism. The stepped terrain of TPG offers comparable but less prominent vertical segmentation and spatial layering, whereas the flat terrain of FPG yields a relatively planar and homogeneous visual scene with limited spatial variation. These spatial interpretations, however, remained speculative, as visual naturalness, spatial hierarchy, visual depth, and enclosure were not objectively quantified in this study. Accordingly, we proposed these landscape characteristics as plausible explanatory hypotheses rather than empirically verified causal factors. Moreover, alternative perceptual differences may also account for the varied restorative effects. MPG provided broader distant views and greater sky visibility (Lin et al., 2023) than TPG and FPG, potentially reducing visual crowding and perceptual fatigue. Variations in scene brightness, color composition, and structural (Wang et al., 2016; Wang et al., 2020; Duan et al., 2024; Hao et al., 2024) complexity across the three VR environments could further modulate subjective restoration and emotional relaxation. In addition, novelty effects and individual preferences for mountainous landscapes may partially explain the stronger perceived restorativeness of MPG, and minor differences in panoramic stitching quality across stimuli cannot be entirely ruled out. Consistent with stress reduction theory (Ulrich et al., 1991), all peony garden scenes elicited significantly better physiological and psychological relaxation than the indoor environment, confirming that vegetation-rich natural settings confer greater restorative benefits than built environments (Ma et al., 2026; Twedt et al., 2016; Tang et al., 2017; Tyrväinen et al., 2014). Collectively, our findings indicated that garden scenes with complex topography, multi-layered spatial structures, and high perceived naturalness correspond to enhanced stress recovery. Nevertheless, definitive causal relationships between specific landscape attributes and restorative outcomes required further empirical validation with quantitative measures of visual features.

This study provides preliminary evidence on the restorative effects of different peony garden VR scenes in college students, thereby extending previous research (Wang et al., 2020; Zhao et al., 2019; Chen, 2015; Guo et al., 2020). Based on peony garden with the different terrain characteristics, we found the MPG showing the stronger physical and mental restoration. Methodologically, the TSST effectively induced acute psychological stress (Ahmad et al., 2017), rather than relying solely on landscape stimulation (Liu L. et al., 2022; Deng et al., 2020; Cao et al., 2024; Malekinezhad et al., 2020; Ma et al., 2026; Chang et al., 2024; Guo et al., 2023; Yakinlar and Akpinar, 2022; Wang et al., 2020; Duan et al., 2024; Hao et al., 2024; Lin et al., 2023; Luo et al., 2022; Korpilo et al., 2024; Li et al., 2025; Qi et al., 2022; Yang et al., 2023). Additionally, VR head-mounted displays delivered immersive, visually realistic simulations (Mattila et al., 2020; Reese et al., 2021), proving reliable for examining landscape-stress relationships (Browning et al., 2019; Wang et al., 2019; Huang et al., 2020; Yu et al., 2020). Collectively, the TSST-VR combination is a valid approach for future studies on specialized plant landscapes (e.g., peony gardens) and human well-being. From a landscape design perspective, these findings offer preliminary perceptual and spatial implications for optimizing campus peony gardens, rather than definitive guidelines. The results suggest that diverse spatial layers, stratified planting layouts, and hierarchical viewing sequences can create visually rich landscapes, while features evoking “being away” and “fascination” may promote psychological and physiological restoration. The distinct configurations of FPG, TPG, and MPG carry different aesthetic and cultural connotations, implying that peony garden design could integrate natural aesthetics with local cultural elements through site-specific strategies for both ornamental and restorative value. However, these implications, derived from VR-based experiments with a student sample, warrant cautious interpretation. Practical factors (accessibility, safety, maintenance, thermal comfort) were not evaluated, and generalizability to broader populations remains untested. Thus, the proposed strategies remain preliminary and require validation via field studies and more diverse population samples.

This preliminary study confirmed that a short-duration VR-based peony garden viewing have favorable stress-relief effects on college students, compared with the indoor control condition. However, several limitations remain. First, the experiment relied solely on VR visual stimulation, ignoring non-visual factors such as peony fragrance, floral morphology (Luo et al., 2022; Huang et al., 2020), garden soundscapes (Evensen et al., 2016), and crowd activities (Lin et al., 2019). Second, only integrated landscape scene types were analyzed. Ecological and environmental indicators (Nian et al., 2010) such as vegetation coverage, green space ratio, peony planting area/density, temperature, humidity, and negative air ion concentration were not systematically examined. Third, the sample was limited to college students, restorative effects may also vary with age and population characteristics (Guo et al., 2023; Yang et al., 2023). The study did not prespecify a primary outcome, and some group comparisons may have insufficient statistical power. The exclusion of female participants reporting menstruation on the test day may have introduced selection bias and reduced female representativeness, given the inconsistent evidence regarding menstrual-cycle effects on cognitive function (Sundström Poromaa and Gingnell, 2014). This criterion also raises concerns regarding sex equity and inclusiveness. Future studies should instead record menstrual-cycle phase, symptoms, and contraceptive use and account for these factors analytically. Fourthly, this study employed only a windowless indoor office as the control condition, without including other natural settings (e.g., urban parks, ordinary gardens, or specialty flower gardens). Thus, our data cannot confirm that peony gardens are superior to other green spaces, nor isolate the specific restorative effects solely attributable to peony plants. Additionally, each landscape type (MPG and TPG) was represented by only a single garden/site. All participants within a group viewed identical stimuli. Thus, adequate participant-level replication does not offset the limited site-level replication across real-world locations. Caution is warranted when generalizing our results to the broader range of landscapes in these categories. Future research should focus on three directions: (1) multi-sensory interactive mechanisms (Li et al., 2025) of peony garden landscapes on physical and mental recovery; (2) coupling relationships between environmental characteristics (physical and ecological factors) and stress restoration outcomes (Nian et al., 2010; Song et al., 2025); (3) differential restorative effects across populations with varying age and occupational backgrounds; and (4) To further assess the comparative restorative potential of peony-themed garden landscapes, we will include more geographically and typologically diverse sites, general urban parks, and other specialty gardens as active control conditions. These efforts will provide theoretical support for health-oriented peony garden design.

## 5 Conclusion

This study combined the TSST for stress induction with VR landscape stimulation to examine the restorative effects of different peony garden landscapes on stress in college students. Results showed that viewing peony garden VR scenes significantly improved students’ physical and mental status, reduced physiological stress indicators and negative emotions, enhanced positive emotions and relaxation, and outperformed the IE. “Being away” and “fascination” were identified as core dimensions of subjective perceived restoration. MPG elicited greater restorative responses than TPG and FPG across multiple indicators, whereas the latter two groups exhibited comparable but measure-specific effects. This research provides a theoretical basis for designing peony-based therapeutic landscapes on campuses in peony-suitable regions.

## Data Availability

The research data utilised to support the findings of this study have not been made public. Requests to access these datasets should be directed to zhaorenlin@xpu.edu.cn.
