# Supplementary material for: Stress recovery and restorative effects of viewing different types of peony garden landscapes: a preliminary experiment
Source: Front Psychol. 2026 Sep 28;17:1935645. doi: 10.3389/fpsyg.2026.1935645 (PMC13654785; doi:10.3389/fpsyg.2026.1935645)
Supplement: Supplementary file 1 [file Table_1.DOCX]

Supplementary Materials

**Supplementary Table 1.** The characteristics of each experimental group's scenarios.

| **Experimental groups** | **Characteristics of peony garden scenes** |
| --- | --- |
| Mountainous peony garden (MPG) | The main elements in the garden include tree peonies, wooden walkways, peony-themed pavilions, hillstone, and mountain steps. The undulating terrain yields a layered three-dimensional view of tree peonies, with slopes, valleys, and ridges creating an alternating open-enclosed visual interface. The coverage rate of tree peonies is 70% to 80%, with an average plant height of 0.55±0.45m. Tree peonies arranged in tiers along the topography, along with forest edges and distant mountains, define three visual planes, resulting in a diverse and dynamically changing landscape. The surroundings are natural mountains and forests. |
| Terraced peony garden (TPG) | This garden is characterized by highland terrace terrain. Its stepped topography creates vertical height differences, allowing layered peony views and local vertical variation. The main elements in the garden include tree peonies, pavilions, natural trails, bamboo and wooden railings, and tile ornaments. The coverage rate of tree peonies is 80% to 90%, with an average plant height of 1.53±0.35m. The surroundings mainly consist of poplars and willows. |
| Flatland peony garden (FPG) | The flat garden lacks vertical relief, presenting a continuous horizontal expanse with minimal depth, resulting in visual monotony and no scene transformation with movement. The main elements in the garden include tree peonies, pavilions, rockeries, winding paths, and wood railings. The coverage rate of tree peonies is 90% to 100%, with an average plant height of 0.80±0.45m. The surroundings mainly consist of plants. |
| Indoor environment (IE) | The office interior measures 8 m (length) × 5 m (width) × 3 m (height), with a floor area of 40 m². It mainly consists of walls, office desks (1.8 × 0.9 m), bookcases, chairs, and hard flooring. The walls and ceiling are off‑white, the floor and desks light yellow, and the chairs black. The office enjoys abundant natural daylight and well‑designed artificial lighting, which ensures sufficient illuminance, uniform light distribution, and freedom from glare or noticeable shadows. Notably, the space is devoid of window landscapes and indoor ornamental plants. |

**Supplementary Table 2.** Comparison of mean values of physiological indexes before and after the Trier Social Stress Test (TSST).

| **Parameter** | **Baseline** | **Stress stage** | **Mean difference** | ***p*** | **95% C.I.** | ***t*** | **Cohen’s *d*** |
| --- | --- | --- | --- | --- | --- | --- | --- |
| HR | 74.48±8.90 | 88.86±9.93 | -14.38 | 0.000^**^ | [-16.35, -12.47] | -14.662 | 1.53 |
| R-R intervals | 0.82±0.11 | 0.70±0.12 | 0.12 | 0.001^**^ | [0.09, 0.14] | 9.917 | 1.04 |
| LF/HF | 2.36±0.76 | 2.58±0.78 | -0.22 | 0.001^**^ | [-0.31, -0.13] | -4.839 | 0.29 |
| HF | 363.09±82.66 | 352.01±79.54 | 11.08 | 0.000^**^ | [5.86, 16.29] | 4.196 | 0.14 |
| Low Alpha | 31115.75±8830.17 | 24694.50±6206.46 | 6421.25 | 0.000^**^ | [4836.86, 8005.75] | 8.011 | 0.84 |
| High Alpha | 24105.75±6478.94 | 19790.25±4663.24 | 4315.50 | 0.000^**^ | [3047.58, 5583.51] | 6.728 | 0.76 |
| Low Beta | 15025.50±3706.86 | 18645.50±4562.20 | -3620.00 | 0.000^**^ | [-4456.10, -2784.02] | -8.559 | 0.87 |
| High Beta | 14360.00±3354.11 | 17633.50±4312.39 | -3273.50 | 0.000^**^ | [-4064.60, -2372.06] | -7.517 | 0.85 |

*n*=224, mean±SD, **p*< 0.05, ***p*< 0.01, paired-sample *t*-test.

**Supplementary Table 3.** Comparison of mean values of physiological indexes between stress stage and recovery stage.

| **Physiological Indices** | **Experimental setting** | **Stress stage** | **Recovery stage** | **Mean difference** | ***p*** | **95% C.I.** | ***t*** | **Cohen’s *d*** |
| --- | --- | --- | --- | --- | --- | --- | --- | --- |
| Heart rate | IE | 87.94±8.04 | 84.78±6.81 | 3.16 | 0.075 | [-0.33, 6.67] | 1.837 | 0.42 |
|  | FPG | 87.15±10.25 | 80.55±8.59 | 6.60 | 0.000^**^ | [4.42, 8.70] | 6.226 | 0.70 |
|  | TPG | 89.46±11.82 | 82.67±8.22 | 6.79 | 0.000^**^ | [3.64, 9.97] | 4.360 | 0.67 |
|  | MPG | 90.90±9.62 | 82.66±4.19 | 8.24 | 0.000^**^ | [5.80, 10.76] | 6.782 | 1.11 |
| R-R intervals | IE | 0.72±0.13 | 0.75±0.10 | -0.03 | 0.160 | [-0.09, 0.02] | -1.436 | 0.00 |
|  | FPG | 0.71±0.12 | 0.77±0.11 | -0.06 | 0.010^**^ | [-0.10, -0.02] | -2.716 | 0.35 |
|  | TPG | 0.68±0.09 | 0.76±0.07 | -0.08 | 0.000^**^ | [-0.12, -0.05] | -4.771 | 0.74 |
|  | MPG | 0.69±0.12 | 0.83±0.12 | -0.14 | 0.000^**^ | [-0.20, -0.10] | -5.493 | 0.75 |
| LF/HF | IE | 2.59±0.73 | 2.33±0.57 | 0.26 | 0.085 | [-0.04, 0.54] | 1.774 | 0.40 |
|  | FPG | 2.50±1.01 | 2.00±1.02 | 0.50 | 0.034^*^ | [0.04, 0.95] | 2.206 | 0.49 |
|  | TPG | 2.57±0.73 | 1.50±0.36 | 1.07 | 0.000^**^ | [0.78, 1.34] | 7.745 | 1.86 |
|  | MPG | 2.66±0.65 | 1.47±0.39 | 1.19 | 0.000^**^ | [0.96, 1.41] | 10.530 | 2.22 |
| HF | IE | 351.43±81.90 | 389.14±84.21 | -37.71 | 0.063 | [-77.64, 2.22] | -1.917 | 0.45 |
|  | FPG | 352.11±75.91 | 399.93±90.07 | -47.82 | 0.013^*^ | [-84.70, -10.96] | -2.633 | 0.57 |
|  | TPG | 363.15±71.92 | 412.44±84.92 | -49.29 | 0.009^**^ | [-85.31, -13.27] | -2.778 | 0.63 |
|  | MPG | 341.35±88.43 | 436.34±108.42 | -94.99 | 0.000^**^ | [-145.03, -44.94] | -3.853 | 0.96 |
| Low Alpha | IE | 27369±4779.00 | 30429±8667.74 | -3060.03 | 0.063 | [-6299.71, 179.65] | -1.918 | 0.44 |
|  | FPG | 22165±6859.99 | 26007±3789.43 | -3841.39 | 0.007^**^ | [-6575.15, -1107.62] | -2.853 | 0.69 |
|  | TPG | 24479±6527.75 | 30844±7643.39 | -6364.61 | 0.000^**^ | [-9090.83, -338.40] | -4.739 | 0.90 |
|  | MPG | 24765±6659.11 | 31639±6660.58 | -6873.47 | 0.000^**^ | [-10076.66, -3670.28] | -4.356 | 1.03 |
| High Alpha | IE | 21090±5189.92 | 24122±6545.10 | -3032.22 | 0.063 | [-6238.39, 173.94] | -1.920 | 0.51 |
|  | FPG | 18197±4583.27 | 25103±7234.58 | -6905.86 | 0.000^**^ | [-9697.71, -4114.01] | -5.002 | 1.14 |
|  | TPG | 18225±4375.17 | 24095±5860.74 | -5869.33 | 0.000^**^ | [-7891.63, -3847.04] | -5.592 | 1.13 |
|  | MPG | 21649±4504.58 | 27303±6564.71 | -5350.22 | 0.002^**^ | [-8682.06, -2018.39] | -3.260 | 1.00 |
| Low Beta | IE | 17948±3095.55 | 16761±3225.28 | 1187.31 | 0.089 | [-190.21, 2564.82] | 1.750 | 0.38 |
|  | FPG | 18037±5457.45 | 15931±2925.57 | 2106.08 | 0.046^*^ | [35.17, 4176.99] | 2.065 | 0.48 |
|  | TPG | 19560±4300.02 | 17257±3906.52 | 2303.39 | 0.040^*^ | [107.43, 4499.34] | 2.129 | 0.56 |
|  | MPG | 19037±5395.76 | 16899±2793.12 | 2137.25 | 0.033^*^ | [181.12, 4093.38] | 2.218 | 0.50 |
| High Beta | IE | 16395±3728.55 | 14921±3025.33 | 1474.50 | 0.082 | [-195.05, 3144.05] | 1.793 | 0.43 |
|  | FPG | 17045±4216.82 | 15556±2759.69 | 1489.36 | 0.049 | [8.44, 2970.28] | 2.042 | 0.42 |
|  | TPG | 18001±4628.09 | 15808±2894.44 | 2193.28 | 0.022^*^ | [332.08, 4054.47] | 2.392 | 0.57 |
|  | MPG | 19093±4676.11 | 16725±3002.89 | 2368.19 | 0.014^*^ | [509.02, 4226.77] | 2.587 | 0.60 |

IE: Indoor environment, FPG: Flatland peony garden, TPG: Terraced peony garden, MPG: Mountainous peony garden. *n*=56, mean±SD, **p*< 0.05, ***p*< 0.01, paired-sample *t*-test.

**Supplementary Table 4.** Comparison of mean values of Psychological indexes between stress stage and recovery stage.

| **Psychological Indices** | **Experimental setting** | **Stress stage** | **Recovery stage** | **Mean difference** | ***p*** | **95% C.I.** | ***t*** | **Cohen’s *d*** |
| --- | --- | --- | --- | --- | --- | --- | --- | --- |
| T-A | IE | 8.53±1.82 | 7.92±1.77 | 0.61 | 0.060 | [-0.03, 1.25] | 1.944 | 0.34 |
|  | FPG | 8.61±2.31 | 6.67±2.24 | 1.94 | 0.000^**^ | [1.70, 2.19] | 16.319 | 0.85 |
|  | TPG | 9.86±2.02 | 7.08±1.75 | 2.78 | 0.000^**^ | [2.52, 3.03] | 21.926 | 1.47 |
|  | MPG | 8.89±1.83 | 6.64±2.18 | 2.25 | 0.000^**^ | [1.80, 2.70] | 10.082 | 1.12 |
| A-H | IE | 8.00±2.08 | 7.86±2.06 | 0.14 | 0.682 | [-0.54, 0.82] | 0.413 | 0.07 |
|  | FPG | 8.06±1.65 | 7.75±1.92 | 0.31 | 0.344 | [-0.34, 0.95] | 0.960 | 0.17 |
|  | TPG | 8.11±3.07 | 6.39±0.86 | 1.72 | 0.003^**^ | [0.64, 2.80] | 3.234 | 0.76 |
|  | MPG | 8.33±2.54 | 6.67±1.39 | 1.67 | 0.001^**^ | [0.71, 2.62] | 3.536 | 0.81 |
| F-I | IE | 8.03±2.54 | 8.42±2.49 | -0.39 | 0.100 | [-0.86, 0.08] | -1.692 | 0.15 |
|  | FPG | 8.11±2.37 | 7.31±2.05 | 0.81 | 0.008^**^ | [0.23, 1.38] | 2.836 | 0.36 |
|  | TPG | 8.17±2.75 | 6.39±1.36 | 1.78 | 0.001^**^ | [0.81, 2.74] | 3.743 | 0.82 |
|  | MPG | 8.22±2.66 | 5.97±1.48 | 2.25 | 0.000^**^ | [1.35, 3.15] | 5.064 | 1.04 |
| D-D | IE | 8.33±2.19 | 8.11±2.13 | 0.22 | 0.316 | [-0.22, 0.67] | 1.016 | 0.10 |
|  | FPG | 9.11±1.87 | 8.19±2.12 | 0.92 | 0.011^*^ | [0.22, 1.61] | 2.686 | 0.46 |
|  | TPG | 8.84±1.99 | 6.50±1.12 | 2.34 | 0.001^**^ | [0.91, 2.97] | 3.823 | 1.45 |
|  | MPG | 9.08±2.01 | 6.81±1.52 | 2.28 | 0.000^**^ | [1.24, 3.32] | 4.443 | 1.27 |
| C-B | IE | 9.02±2.29 | 8.48±2.28 | 0.54 | 0.078 | [-0.08, 1.35] | 1.814 | 0.24 |
|  | FPG | 9.25±2.26 | 7.39±2.31 | 1.86 | 0.000^**^ | [1.50, 2.22] | 10.406 | 0.81 |
|  | TPG | 9.92±1.28 | 7.64±2.25 | 2.28 | 0.000^**^ | [1.95, 2.61] | 14.028 | 1.25 |
|  | MPG | 9.89±1.08 | 6.75±2.21 | 3.14 | 0.000^**^ | [2.63, 3.65] | 12.433 | 1.81 |
| V-A | IE | 20.08±4.17 | 20.36±4.33 | -0.28 | 0.402 | [-0.94, 0.38] | -0.848 | 0.07 |
|  | FPG | 19.83±3.65 | 20.69±3.88 | -0.86 | 0.002^**^ | [-1.39, -0.33] | -3.290 | 0.23 |
|  | TPG | 18.97±3.73 | 21.94±5.18 | -2.97 | 0.004^**^ | [-4.94, -1.01] | -3.073 | 0.70 |
|  | MPG | 18.08±5.54 | 20.81±4.64 | -2.72 | 0.000^**^ | [-4.11, -1.34] | -3.988 | 0.53 |
| STAI-S | IE | 44.50±8.27 | 41.14±8.38 | 3.36 | 0.084 | [-0.47, 7.20] | 1.781 | 0.40 |
|  | FPG | 45.36±11.56 | 33.64±8.07 | 11.72 | 0.000^**^ | [8.66, 14.88] | 7.545 | 1.18 |
|  | TPG | 48.72±9.63 | 35.97±8.10 | 12.75 | 0.000^**^ | [8.18, 17.32] | 5.669 | 1.43 |
|  | MPG | 52.11±8.95 | 36.25±9.06 | 15.86 | 0.000^**^ | [11.96, 19.76] | 8.249 | 1.76 |

T-A: tension-anxiety, A-H: anger-hostility, F-I: fatigue-inertia, C-B: confusion-bewilderment, D-D: depression-dejection, and V-A: vigor-activity. IE: Indoor environment, FPG: Flatland peony garden, TPG: Terraced peony garden, MPG: Mountainous peony garden. *n*=56, mean±SD, **p*< 0.05, ***p*< 0.01, paired-sample *t*-test.
